# Non-equivalence of sub-tasks of the Rey-Osterrieth Complex Figure Test with convolutional neural networks to discriminate mild cognitive impairment

**DOI:** 10.1186/s12888-024-05622-5

**Published:** 2024-02-27

**Authors:** Jin-Hyuck Park

**Affiliations:** Department of Occupational Therapy, College of Medical Science, Soonchunhyang University, Room 1401, College of Medical Science, 22 Soonchunhyang-ro, Shinchang-myeon, Asan, Chungcheongnam-do 31538 Republic of Korea

**Keywords:** Screening, Convolutional neural network, Rey-Osterrieth Complex Figure Test, Mild cognitive impairment

## Abstract

**Background:**

The Rey-Osterrieth Complex Figure Test (RCFT) is a tool to evaluate cognitive function. Despite its usefulness, its scoring criteria are as complicated as its figure, leading to a low reliability. Therefore, this study aimed to determine the feasibility of using the convolutional neural network (CNN) model based on the RCFT as a screening tool for mild cognitive impairment (MCI) and investigate the non-equivalence of sub-tasks of the RCFT.

**Methods:**

A total of 354 RCFT images (copy and recall conditions) were obtained from 103 healthy controls (HCs) and 74 patients with amnestic MCI (a-MCI). The CNN model was trained to predict MCI based on the RCFT-copy and RCFT-recall images. To evaluate the CNN model’s performance, accuracy, sensitivity, specificity, and F1-score were measured. To compare discriminative power, the area under the curve (AUC) was calculated by the receiver operating characteristic (ROC) curve analysis.

**Results:**

The CNN model based on the RCFT-recall was the most accurate in discriminating a-MCI (accuracy: RCFT-copy = 0.846, RCFT-recall = 0.872, MoCA-K = 0.818). Furthermore, the CNN model based on the RCFT could better discriminate MCI than the MoCA-K (AUC: RCFT-copy = 0.851, RCFT-recall = 0.88, MoCA-K = 0.848). The CNN model based on the RCFT-recall was superior to the RCFT-copy.

**Conclusion:**

These findings suggest the feasibility of using the CNN model based on the RCFT as a surrogate for a conventional screening tool for a-MCI and demonstrate the superiority of the CNN model based on the RCFT-recall to the RCFT-copy.

## Background

Neuropsychological assessments play a crucial role in achieving an objective diagnosis for patients with cognitive impairment [1]. Among various neuropsychological assessments, the Rey-Osterrieth Complex Figure Test (RCFT) has been widely employed to assess cognitive function. Notably, the RCFT has proven valuable for analyzing visuospatial construction, perceptual organization, and visual memory in clinical evaluations and research studies [2].

The RCFT includes immediate copy and delayed recall tasks. During the copy task of the RCFT, subjects are required to visually examine a complex geometric figure and then replicate it as accurately as possible on a blank sheet of paper. This task assesses their visuo-constructional skills and executive function, which involve the ability to perceive and accurately reproduce visual information [3]. The second task of the RCFT is the delayed recall. At 30 min after completing the copy task, subjects are asked to draw the figure again from memory, without any visual reference. This task evaluates their visual memory, specifically their ability to retain and recall complex visual information after a delay [4]. Both copy and recall performance are evaluated based on the accuracy and organization of the reproduced figure using scoring criteria, including accuracy, organization, and placement of the different components of the figure [4].

A previous study reported that patients in the early stages of cognitive impairment often exhibit poor performance on the RCFT [5, 6]. Specifically, patients with frontal lobe damage demonstrate an impairment in their abilities to reproduce the Rey-Osterrieth Complex Figure [7]. Given the importance of identifying and diagnosing mild cognitive impairment (MCI) at an early stage, there is a growing interest in the potential of using the RCFT to detect MCI. Indeed, prior studies have reported that the RCFT can significantly predict the conversion to pre-MCI or MCI as patients with MCI commonly show executive dysfunction and memory declines [8].

Although the RCFT has scoring criteria, it has some inherent subjectivity that can influence its results and interpretation, leading to challenges in the detection of MCI.^6^ To address this issue, recently there have been studies reporting the use of convolutional neural network (CNN) techniques to analyze images of the RCFT. These studies demonstrated the clinical feasibility of the RCFT using CNN techniques [6, 9, 10]. Moreover, a CNN-based approach has clinical significance as it allows for automated evaluation of the RCFT, potentially saving time and effort in analyzing large amounts of data.

A previous work using CNN techniques for the RCFT to discriminate MCI utilized the RCFT’s copy condition alone [6]. However, the potential utility of a CNN model based on the RCFT’s recall condition remains unclear despite the confirmed benefits of a CNN model using the RCFT’s copy condition in discriminating MCI [6]. Considering individuals with MCI show both executive function and memory deficits depending on the subtype of MCI [11, 12], the clinical feasibility of the RCFT’s recall condition also needs to be examined. Furthermore, as memory impairment is a stronger predictor of progression from MCI to Alzheimer’s disease than impairment in other cognitive domains [13], it is worthwhile to apply the RCFT’s recall condition to the amnestic type of MCI (a-MCI), a subtype of MCI that exhibits predominant memory decline. Indeed, it has been acknowledged that the previous study’s findings have a limitation attributed to its exclusive focus on analyzing the copy condition, which further supports the need to evaluate the feasibility of a CNN model based on the RCFT’s recall condition for the detection of a-MCI. Consequently, it is necessary to compare the difference in CNN model performance between conditions of the RCFT in discriminating a-MCI.

Therefore, this study utilized the CNN model based on both RFCT’s copy and recall to distinguish a-MCI from healthy aging. The primary aim of this study was to investigate whether the RCFT with CNNs could be employed as a screening tool for MCI. This study also sought to compare the discriminant power of CNN models between the RCFT’s copy and recall.

## Methods

### Design

This study employed an observational study design using data from the author’s previous study examining the feasibility of the newly developed screening system for MCI in South Korea. This study was approved by the Institutional Review Board of Yonsei University (1041849-201611-BM-060-01). All subjects provided informed consent before participating in the present study according to the Declaration of Helsinki (2004).

### Dataset

The original data set from the author’s previous study consisted of 103 healthy controls (HCs) and 74 patients with amnestic MCI (a-MCI) [11]. All subjects were older than 65 years. They were recruited from communities and welfare centers in South Korea. The HC group consisted of 103 subjects without memory complaints. They were in the normal range for the standardized neuropsychological battery, the Seoul Neuropsychological Screening Battery. The MCI group consisted of 74 individuals with a-MCI defined according to a previous study [13]. Inclusion criteria were as follows: (a) subjective memory complaint, (b) objective memory impairment relative to age- and education-matched HCs confirmed by a score on the Seoul Verbal Learning Test (below 1.5 standard deviations), (c) intact general cognitive function confirmed by the Korean version of the Mini-Mental State Examination (MMSE-K) score (≥ 24), and (d) intact activities of daily living as identified by score on Seoul instrumental activities of daily living score ≤ 7. Exclusion criteria were as follows: (a) prior diagnosis of dementia by physicians, (b) presence of neurological or psychiatric disorders (e.g., stroke or schizophrenia), (c) moderate to severe depressive symptoms defined by a score on the Beck Depression Scale, and (d) any auditory or visual impairments.

### Measurement

In the author’s previous study [10], the RCFT was implemented by trained occupational therapists. Subjects were given an A4-sized paper and a pencil and asked to copy the presented Rey Complex Figure (RCFT-copy). 30 min after the copy condition, they were instructed to draw the figure again on another A4-sized paper, relying on their memory (RCFT-recall).

### Data preprocessing

A total of 354 RCFT images (copy and recall conditions) were obtained from 104 HCs and 74 patients with MCI. Since the original RCFT samples were not suitable to impute to a CNN model directly as the size and direction of the figure varied across subjects, raw samples were preprocessed by automatically cropping in a square and resized to 224 × 224 regardless of the raw sample size. In this process, all samples were placed in a 600-dpi template and saved in the “.png” format. In addition, all images were converted into tensors and subsequent normalization. The pixel values of the images were scaled to a range between 0 and 1 by dividing them by 255 for normalization. Subsequently, the preprocessed samples were augmented to increase the number of samples to impute to the CNN model by adding a combination of the following manipulation according to a previous study: rotation (90 or 180 degrees), horizontal flip, or vertical flip, resulting in a total of 708 samples [6]. For test samples, only original samples were used.

### CNN structure

In this study, a CNN with four convolutional layers including max pooling layers after each convolutional layer and two fully connected layers was adopted. To avoid overfitting, a drop layer was introduced between fully connected layers. There were 32 convolutions with 3 × 3 kernels in each layer. The two fully connected layers had 256 and 128 neurons, respectively. A rectified linear unit (ReLU) function was used as an activation function. This CNN model was established in accordance with a previous study [14].

### Model training and validating

For training a CNN model, the experiment was implemented in Python using the Keas package with Tensorflow. Model training was performed to increase accuracy and its validation for a maximum epoch of 30 times. The batch size was set to 32. To maximize the validating process, early stopping was arbitrarily applied based on the validation accuracy curve. Binary cross-entropy was used as a loss function. For minimizing or maximizing the loss function, Adam optimizer was used with its default setting in Keras. A 5-fold cross-validation was used. Subsequently, the trained CNN model was applied to the test sub-group to evaluate the CNN model’s performance. Standard metrics (accuracy, sensitivity, specificity, and F1-score) were measured.

### Statistical analysis

SPSS for Windows (version 22.0) was used to analyze data in this study. The general and clinical characteristics of subjects were analyzed using descriptive statistics. The area under the curve (AUC) was calculated by performing a Receiver Operating Characteristic (ROC) curve analysis.

## Results

### Basic features

Demographic and clinical characteristics of datasets included sex, age, education period, and the Korean version of the Montreal Cognitive Assessment (MoCA-K) scores. There was no significant difference in sex ratio, age, and education period between the HC and a-MCI groups (*p*’s > 0.05) (Table 1). However, a significant difference was found in the MoCA-K score between both groups, with the a-MCI group showing a lower cognitive function than the HC group (*p* < 0.05) (Table 1).

**Table 1:** General characteristics of participants (*N* = 177)

Characteristics	a-MCI (*n* = 74)	NC (*n* = 103)	χ^2^ / t	*p*
Age, years (SD)	74.45 (6.51)	74.93 (6.96)	.471	0.639
Sex. N (%)				
Male	33 (44.6)	45 (43.7)	.905	0.513
Female	41 (55.4)	58 (56.3)		
Education, years (SD)	6.14 (4.53)	5.83 (4.52)	-.450	0.654
MoCA-K, scores (SD)	22.89 (2.17)	25.74 (2.10)	9.894	<0.001

*MoCA-K *Korean version of the Montreal Cognitive Assessment

### Classification performance

**Fig. 1:**
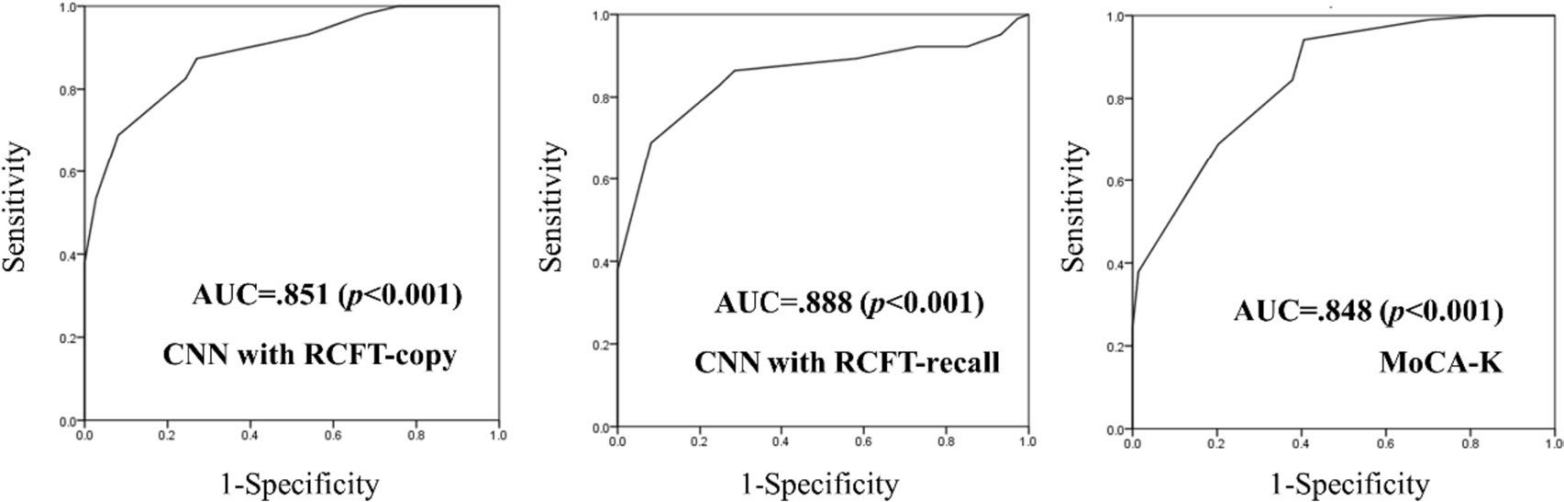
ROC curves of three predictors. Greater AUC values indicate higher power in discriminating a-MCI from HCs

During the CNN analysis, an overfitting was not found. Overall performance in the test sub-group is summarized in Table 2. The CNN model based on the RCFT-recall was more accurate in discriminating a-MCI from HCs than the CNN model based on the RCFT-copy and the MoCA-K (recall: 0.853; copy: 0.824; MoCA-K: 0.818). Specifically, the CNN model based on the RCFT-recall achieved the highest specificity of 0.854 whereas, the MoCA-K showed the highest sensitivity of 0.951. Furthermore, the CNN model based on RCFT-recall achieved the highest AUC value, followed by the RCFT-copy (Fig. 1). These findings suggest that the CNN model with the RCFT can better discriminate a-MCI than the MoCA-K and that the CNN model based on the RCFT-recall has higher discriminative power for a-MCI than the RCFT-copy.

**Table 2:** Convolutional neural network performance and the MoCA-K for detection of a-MCI

Features	Accuracy	Sensitivity	Specificity	F1-score
RCFT				
Copy condition	0.846	0.843	0.851	0.864
Recall condition	0.872	0.864	0.884	0.890
MoCA-K, scores	0.818	0.951	0.630	0.859

*MoCA-K *Korean version of the Montreal Cognitive Assessment, *RCFT *Rey Complex Figure Test

## Discussion

This study investigated the feasibility of using the CNN model based on the RCFT as a screening tool for a-MCI and compared the non-equivalence of sub-tasks (copy and recall conditions) of the RFCT. The findings of this study revealed that the CNN model based on the RCFT-recall was more accurate in differentiating MCI than the RCFT-copy and the MoCA-K, suggesting the superiority of the RCFT-recall in distinguishing a-MCI.

In the RCFT-copy, the complexity of the figure requires an ability to organize it into a meaningful perceptual unit. It involves cognitive processes related to executive function [15], which is frequently impaired in patients with MCI. Specifically, regardless of the sub-types of MCI, patients with MCI, even those with pure a-MCI, were significantly more impaired than HCs in neuropsychological assessments of executive function [16]. These findings support that the RCFT-copy can sufficiently differentiate a-MCI [6]. Similarly, this study found that RCFT-copy achieved satisfactory sensitivity and specificity in detecting a-MCI from HCs with a high accuracy, which is in line with previous findings [6, 15, 16].

Contrary to a previous study that only examined the RCFT-copy [6], this study investigated both the RCFT-copy and RCFT-recall to confirm their non-equivalence for distinguishing a-MCI. This study revealed that the CNN model based on the RCFT-recall was superior to the RCFT-copy in differentiating a-MCI, which is consistent with the findings of a previous study [17]. Furthermore, a previous study reported that the RCFT-copy alone is insufficient in screening MCI [18], supporting the current findings. This non-equivalence could be attributed to the characteristics of patients with a-MCI in this study. Since patients with a-MCI were selected out of those with sub-types of MCI in this study, visual memory assessed by the RCFT-recall might be more representative of a-MCI’s cognitive traits than executive function assessed by the RCFT-copy. This could affect the superiority of the RCFT-recall to differentiate a-MCI [17]. Indeed, patients with a-MCI are distinctly characterized by memory impairment [19]. Accordingly, the superiority of the RCFT-recall to detect MCI might not necessarily be confirmed in other types of MCI. Nevertheless, RCFT-recall is still important for distinguishing a-MCI from HCs, with the exception of non-amnestic MCI as patients with MCI have common memory deficits [20]. It was interesting to note that despite the superiority of the RCFT-recall, even the RCFT-copy had higher discriminative power than the MoCA-K for a-MCI. This discovery suggests that the RCFT-copy, assessing a single cognitive domain, might prove valuable in the screening of a-MCI compared to the MoCA-K, which evaluates multiple cognitive domains.

Notably, both the CNN model based on the RCFT-recall and that based on the RCFT-copy had higher specificity than the MoCA-K, demonstrating their advantages as screening tools. High specificity means that it is good at identifying individuals who do not have a particular condition or disease. In other words, high specificity can ensure fewer false positives. False positives can lead to unnecessary further testing. Thus, reducing false positives allows for more efficient use of a screening tool [21]. On the other hand, the MoCA-K showed the highest sensitivity. The high specificity of the CNN model based on the RCFT-recall and the high sensitivity of the MoCA-K might provide the optimal combination in differentiating a-MCI from HCs by minimizing false positives and false negatives, respectively.

On the other hand, due to the inherent limitations of CNNs, this study was unable to specify which visual features of the RCFT were utilized to discriminate a-MCI. Nevertheless, in paper-based RCFT literature, there is a recognition of the challenge associated with limited visual analysis for identifying the visual features of the RCFT. The complexity of the RCFT exacerbates the difficulties associated with visual analysis, thereby steering us towards a black box deep learning approach to surmount these challenges [22]. Indeed, errors in specific parts of the RCFT are not consistently observed in patients with MCI. Therefore, the lower accuracy of the figures, rather than the features of the figures, emerges as a pivotal factor in the MCI screening process [4].

Meanwhile, the variation in the strictness applied by different experts is evident, and the inter-rater score differences, reaching up to 20%, substantiate this disparity [22]. Consequently, attempts have been made to achieve objective scoring through the utilization of automatic scoring techniques using machine learning. However, this approach may inadvertently introduce potential biases, as it fails to consider the contextual nuances of subjects and relies on a standardized assessment. Therefore, even automatic scoring techniques are not a complete replacement for CNNs.

Several studies have demonstrated that a digital version of a paper-based screening tool is capable of differentiating patients with cognitive impairment from HCs using machine learning [6, 14, 21, 23, 24]. It should have greater potential than a conventional paper-based tool in terms of its capability to collect large amounts of data quickly. In line with this trend, the digital version of the RCFT could be popularized to obtain a large amount of data from patients with MCI. Nevertheless, a digital RCFT requires machine learning techniques to analyze a great quantity of data. Therefore, the findings of this study, affirming the feasibility of deep learning models based on the RCFT to screen a-MCI, have clinical implications. Subsequently, digital RCFT with classification models can be used to remotely monitor the cognitive status of community residents. Moreover, its high specificity can reduce false alarms of cognitive decline [25], suggesting that it has more potential than the original RCFT.

This study has some limitations. Firstly, in contrast to previous studies using publicly distributed data [14], the small sample size of this study might limit the generalizability of its findings. Furthermore, it is impossible to rule out the influence of outliers due to a small sample size. Nevertheless, this study clearly demonstrated the feasibility of the CNN approach with the RCFT to distinguish MCI by enrolling patients with MCI based on the standard MCI criteria, compared to previous studies that determined MCI based on the Clinical Dementia Rating alone [6]. Moreover, considering that the standard deviation of the MOCA-K scores of subjects in each group was not substantial, outliers may not have significantly distorted the results of this study. Secondly, since the subjects of this study were limited to a-MCI, its findings could not be generalized to other types of MCI. However, considering that most previous studies commonly involved a-MCI because patients with a-MCI show minimal cognitive bias of a-MCI and they strongly predispose subjects toward Alzheimer’s disease [11, 12, 26], the current findings have clinical implications. Thirdly, even though the RCFT is superior in discriminating patients with a-MCI compared to the MoCA-K, it is recommended not to rely solely on the RCFT, as it may not encompass all cognitive domains. Fourthly, since no effort was made to find an optimized CNN model to improve its accuracy, the accuracy could be higher. Therefore, the findings of this study should be interpreted with caution. Nonetheless, since the purpose of this study was to explore the feasibility of using the CNN model based on the RCFT for MCI screening and determine the non-equivalence between the RCFT-recall and RCFT-copy, model optimization studies with a large amount of data are needed in the future.

## Conclusion

In the current study, the CNN model based on the RCFT achieved similar performance to previous studies in terms of accuracy with subjects based on MCI criteria, suggesting its feasibility for detecting MCI. Moreover, the superiority of the CNN model based on the RCFT-recall to the RCFT-copy was found. Since the CNN model based on the RCFT possesses some advantages, such as the availability of considerable accumulated past data and greater accessibility than the original RCFT, it could be a surrogate for a clinical-based screening tool for MCI.

## Data Availability

The data presented in this study are available on request from the corresponding author.
